# A High-Performance Liquid Chromatography with Photodiode Array Detection Method for Simultaneous Determination of Three Compounds Isolated from *Wikstroemia ganpi*: Assessment of the Effects on Cytochrome P450-Mediated Metabolism In Vitro and In Vivo

**DOI:** 10.3390/nu15184061

**Published:** 2023-09-19

**Authors:** Min-Ji Keem, Seong-Wook Seo, Taeyoung Kim, Beom-Geun Jo, Su-Nam Kim, In-Soo Yoon, Min Hye Yang

**Affiliations:** 1 Department of Pharmacy, College of Pharmacy, Pusan National University, Busan 46241, Republic of Korea; mj_keem@pusan.ac.kr (M.-J.K.); bg_jo@pusan.ac.kr (B.-G.J.); 2 Research Institute for Drug Development, Pusan National University, Busan 46241, Republic of Korea; sswook@pusan.ac.kr (S.-W.S.); taeyk@pusan.ac.kr (T.K.); 3 Department of Manufacturing Pharmacy, College of Pharmacy, Pusan National University, Busan 46241, Republic of Korea; 4 Natural Products Research Institute, Korea Institute of Science and Technology, Gangneung 25451, Republic of Korea; snkim@kist.re.kr

**Keywords:** *Wikstroemia ganpi*, 7-methoxylutolin-5-*O*-glucoseide, herb–drug interaction, HPLC-PDA, pharmacokinetics, pilloin 5-*O*-β-d-glucopyranoside, rutarensin, validation

## Abstract

In natural products, the content and quality of the marker components differ depending on the part, production area, collection period, and extraction method; therefore, a standardized analysis method is required to obtain consistent results. This study developed a simultaneous analysis method for three marker components (7-methoxylutolin-5-*O*-glucoseide, pilloin 5-*O*-β-d-glucopyranoside, rutarensin) isolated and purified from *Wikstroemia ganpi* (*W. ganpi*). Simultaneous analysis was performed using high-performance liquid chromatography with photodiode array detection (HPLC-PDA) method that was validated according to the International Council for Harmonisation (ICH) guidelines. The developed analytical method exhibited linearity (*r*^2^ > 0.999), detection limits (0.72–3.34 μg/mL), and quantification limits (2.19–10.22 μg/mL). The relative standard deviation (RSD) value of intra- and inter-day precisions was less than 1.68%, and analyte recoveries (93.42–117.55%; RSD < 1.86%) were validated according to the analytical procedures, and all parameters were within the allowable range. Quantitative analysis of the three marker components from *W. ganpi* MeOH extract (WGM) showed 7-methoxylutolin-5-*O*-glucoseide with the highest content (51.81 mg/g). The inhibitory effects of WGM on cytochrome P450 (CYP) substrate drugs were further investigated. The in vitro study revealed that WGM inhibited the CYP3A-mediated metabolism of buspirone and that 7-methoxylutolin-5-*O*-glucoseide and pilloin 5-*O*-β-d-glucopyranoside inhibited the metabolism of buspirone with IC_50_ values of 2.73 and 18.7 μM, respectively. However, a single oral dose of WGM did not have significant effects on the pharmacokinetics of buspirone in rats, suggesting that WGM cannot function as an inhibitor of CYP3A-mediated metabolism in vivo.

## 1. Introduction

*Wikstroemia ganpi* (Siebold & Zucc.) Maxim. (*W. ganpi*) is mainly distributed in Korea, Japan, and Taiwan. It is known as “geo-mun-do-dak-na-mu” in Korean and “ko-ganpi” in Japanese [1]. *W. ganpi* is a member of the *Thymelaeaceae* family [2], and its synonyms are *Diplomorpha ganpi* (Siebold & Zuccarini) Nakai, *Passerina ganpi* (Siebold & Zucc.), and *Stellera ganpi* (Siebold & Zucc.) Meisn. According to previous studies, plants in the genus *Wikstroemia* contain various physiologically active substances, including flavonoids [3,4], coumarins [5], and lignans [6], with anti-inflammatory [7,8], anti-cancer [9,10], and antifungal activities [11]. Recently, a study reported that flavonoid components obtained by separating the roots of *W. ganpi* have anti-atopic activity [12].

Phenolic compounds, which are secondary plant metabolites, are categorized according to their chemical structure into simple phenols, phenolic acids, flavonoids, tannins, and coumarins [13]. They are known to have antioxidant, antibacterial, and anti-allergic properties and to prevent hyperlipidemia [14]. Flavonoids are phenolic compounds composed of two benzene and heterocyclic rings, which are known to inhibit the rate of oxidation and free radical formation via intracellular lipoxygenase activity [15]. It has also been reported that cardiovascular diseases can be improved with their use, and there are also anti-cancer, antioxidant, and anti-inflammatory effects of flavonoids [16]. Another phenolic compound, coumarin, is a heterocyclic compound belonging to the class of benzopyrones [17]. Compounds with a coumarin skeleton have anti-inflammatory effects and are reported to be inhibitory in α-chymotrypsin and human leukocyte diastase [18]. Natural products contain various substances other than pharmacological substances, and their composition ratios vary depending on habitat, climate, and site [19]. Therefore, it is necessary to standardize the quality, purity, and reliability of analyte targets. Nuclear magnetic resonance (NMR) spectroscopy and mass spectrometry (MS) are primarily used for the qualitative analysis of chemicals. NMR spectroscopy is less sensitive than MS spectroscopy but is more effective in identifying and quantifying metabolites in tissue extracts [20]. The chemical profile can be used to analyze substances using chromatographic fingerprints such as high-performance liquid chromatography (HPLC) [21], gas chromatography (GC) [22], and capillary electrophoresis (CE) [23], and spectral fingerprints such as ultraviolet (UV), infrared (IR), and mass spectrometry (MS) [24,25]. In particular, HPLC equipped with photodiode array (PDA) detectors has the advantage of being able to use a variety of solvents and is the most commonly used liquid chromatography method for material analysis. It is also a certified method for the overall quality assessment of drugs [26].

The analytical methods used for natural products should be validated (quality, safety, efficacy, etc.), and the International Council for Harmonisation (ICH) guidelines are appropriate for this [27]. The ICH guidelines discuss the characteristics that should be considered when validating analytical procedures and are used to demonstrate the suitability of a developed analytical procedure for its intended purpose. Several countries refer to ICH Q2(R1) guidelines [28] because the statistical approach used in the validation of methods maximizes the efficiency of a validation test. The data presented for the optimal design of the test methods were as follows: specificity, linearity, range, accuracy, precision, and recovery. Due to insufficient phytochemical studies and data on marker components, standardization of the method is important for obtaining accurate and reliable results for future experimental data. Therefore, for the standardization of *W. ganpi*, the chemical structures of three marker compounds (two flavonoids and one coumarin) isolated from the methanol (MeOH) extract were identified using NMR, and the HPLC-PDA analysis was developed and validated according to the ICH guidelines.

Herb–drug interactions (HDIs) are increasingly being recognized as important clinical factors that may alter the oral bioavailability of therapeutic agents co-administered with herbal medicines [29]. Many of the major pharmacokinetic interactions between drugs are due to the effects of previous drug administration on hepatic cytochrome P450 (CYP) enzymes [30]. In particular, the modulation of drug metabolic enzymes, such as CYP induction or inhibition, via herb-derived compounds is one of the well-recognized primary causes of HDIs [31,32]. Several studies have shown that herbal medicines can inhibit CYP activity, causing serious HDIs when combined with conventional medicines [33]. To the best of our knowledge, in vitro studies have evaluated the biological activity of *W. ganpi* MeOH extract (WGM), but no studies have evaluated the HDI potential of WGM itself. Therefore, the evaluation of HDIs associated with drug-metabolizing enzymes is necessary to ensure the safe use of herbal products. Herein, we report the direct effects of WGM and marker compounds on CYP-mediated drug metabolism in rats in vitro and in vivo. The inhibitory effect of the three marker compounds of WGM on CYP metabolic activity in the rat liver S9 fraction was evaluated for their half-maximal inhibitory concentrations (IC_50_). The in vivo pharmacokinetic interaction was evaluated following a single oral administration of buspirone (BUS; probe substrate for CYP3A) [34,35] alone or co-administered with WGM in a rat model.

## 2. Materials and Methods

### 2.1. Materials and Animal

ACS/HPLC reagent-grade acetonitrile, methanol, and water were procured from Honeywell (Morristown, NJ, USA). HPLC-grade dimethyl sulfoxide (DMSO) and formic acid (FA) were procured from Junsei Chemical Co., Ltd. (Tokyo, Japan) and Dae-Jung Chemicals and Metals Co., Ltd. (Siheung, Republic of Korea). Buspirone (BUS), dextromethorphan (DEX), diclofenac (DIC), and alpelisib (IS) were procured from MedKoo Bioscience, Inc. (Morrisville, NC, USA). Magnesium Chloride (MgCl_2_), β-nicotinamide adenine dinucleotide 2′-phosphate reduced tetrasodium salt hydrate (NADPH), polyethylene glycol 400 (PEG400), and potassium phosphate buffer were procured from Sigma-Aldrich Co. (St. Louis, MO, USA). Sprague Dawley (SD) rat liver S9 fraction was procured from Sekisui Xenotech (Kansas City, KS, USA). SD rats (Male; nine-week-old; weighing range: 250–270 g) were supplied by Koatech Co. (Pyeongtaek, Republic of Korea). The SD rats were used for pharmacokinetic studies after an acclimation period of 7 days. Feeding, excluding water, was restricted for 12 h before starting the pharmacokinetics study. All animal studies were conducted with approval from the Institutional Animal Care and Use Committee of Pusan National University (approval number: PNU-2023-3245).

### 2.2. Plant Materials

*W. ganpi* were collected from Geumsa-ri, Yeongnam-myeon, Goheung-gun, Jeollanam-do, Republic of Korea, and the aerial parts were air-dried for use. They were identified by Jin-Hyub Paik (International Biological Material Research Center, Korea Research Institute of Bioscience and Biotechnology, Daejeon, Republic of Korea). A voucher specimen marked as PNU-0027 has been deposited at the College of Pharmacy of Pusan National University (Busan, Republic of Korea).

### 2.3. Extraction and Isolation

Air-dried aerial parts of *W. ganpi* (4.27 kg) were extracted two times for 90 min with 30 L of MeOH using an ultrasonic extractor and filtered. After the filtrates were mixed and evaporated in a vacuum below 40 °C, a semisolid residue was obtained. It was then freeze-dried to produce WGM at an 11.3% yield (483.0 g). The WGM was suspended in distilled water and subsequently partitioned with *n*-hexane (4 L), ethyl acetate (4 L), and *n*-butanol (4 L). The *n*-butanol fraction (WGB, 122.6 g) was chromatographed on silica gel (70–230 mesh ASTM) and eluted using a gradient system with CH_2_Cl_2_-MeOH (30:1 → 100% MeOH), and 10 fractions (WGB1–WGB10) were collected. WGB8 was fractionated into 6 subfractions (WGB8-1–WGB8-6) using silica gel column chromatography with CH_2_Cl_2_-MeOH (10:1 → 100% MeOH). The insoluble fraction of WGB8-6 was recrystallized from MeOH and yielded compound **1** (38.0 mg). WGB7 was fractionated into 10 fractions (WGB7-1–WGB7-10) using silica gel column chromatography eluted using isocratic elution with CH_2_Cl_2_-MeOH (10:1). Subfraction WGB7-6 was recrystallized from MeOH and compound **2** (638.7 mg) was obtained. WGB10 was fractionated into 10 subfractions (WGB10-1–WGB10-10) using silica gel column chromatography with CH_2_Cl_2_-MeOH (10:1 → 100% MeOH). The insoluble fraction of WGB10-10 was fractionated into 7 subfractions (WGB10-10-1–WGB10-10-7) using silica gel column chromatography with CH_2_Cl_2_-MeOH (10:1 → 100% MeOH). WGB10-10-7 subfraction was separated by semi-preparative HPLC (INNO C_18_, 10 μm particle size, 10 mm × 250 mm; detection, UV at 254 nm; flow rate, 2 mL/min) using a gradient solvent system of 0.1% FA-acetonitrile/0.1% FA-water (27.5:72.5, *v*/*v*) led to isolation of compound **3** (61.2 mg, *t*_R_ = 33.5 min). The molecular structures of the isolated compounds from WGM were identified by comparing the spectroscopic analysis results with the references.

### 2.4. Preparation of Sample and Reference Solutions

Powdered WGM was dissolved in DMSO/MeOH (50:50, *v*/*v*) at a concentration of 20.0 mg/mL and used as the sample stock solution. Reference compounds [7-methoxyluteolin-5-*O*-glucoside (**1**), pilloin 5-*O*-β-d-glucopyranoside (**2**), and rutarensin (**3**)] isolated from WGM were dissolved in DMSO/MeOH (50:50, *v*/*v*) at a concentration of 1200 µg/mL and used as reference stock solutions. Stock solutions were refrigerated at 4°C, and working solutions were prepared and tested every time.

### 2.5. Determination of HPLC-PDA Conditions

Analysis was performed using a Waters HPLC-PDA system (Waters Co., Milford, MA, USA) consisting of an e2695 separation module and a 2998 photodiode array (PDA) detector. The control of chromatographic processing and data acquisition was performed using Empower^®^ 3 Software. Aegispak C_18_-L column (5 µm, 4.6 mm × 250 mm) was used (30 °C). The eluents consisted of 0.1% FA in acetonitrile (A) and 0.1% FA in water (B), as follows: 0.0–5.0 min, 13–20% A; 5–23 min, 20–30% A; 23–25 min, 30–70% A; 25–30 min, 70% A. (flow rate: 1 mL/min). The UV wavelength of the detector was set to 340 nm, and 10 μL of the samples were injected into the HPLC-PDA system for analysis.

### 2.6. HPLC-PDA Method Validation

The developed HPLC-PDA simultaneous analysis conditions were validated according to ICH Q2(R1) guidelines [36]. The validation characteristics included specificity, linearity, detection limit, quantitation limit, precision, recovery, and stability. Specificity is defined as the ability to clearly identify an analyte in a sample containing a mixture of impurities. This was verified by comparing and overlaying the chromatograms and PDA spectra obtained from the WGM samples and the reference mixture to confirm that the retention time (*t*_R_), spectral pattern, and maximum absorption wavelength (λ_max_) of the three reference compounds were consistent. Linearity is a test used to derive calibration curves by obtaining measurements that are directly proportional to the concentration of the substance being examined within a certain range. The stock solutions of 7-methoxyluteolin-5-*O*-glucoside (**1**), pilloin 5-*O*-β-d-glucopyranoside (**2**), and rutarensin (**3**) were diluted to six target concentrations (5.0, 50.0, 250.0, 400.0, 500.0, and 600.0 μg/mL) in DMSO/MeOH (50:50, *v*/*v*) mixtures, and six replicates (*n* = 6) were performed for each working solution. The calibration curve was expressed using a linear equation (y = ax + b) through linear regression analysis as a function of the area (y) vs. the concentration (x) of each reference peak, and the slope (a), y-intercept (b), and correlation coefficient (*r*^2^) were calculated. The correlation coefficients for the acceptance criteria exceeded 0.999. The detection limit (DL) and quantitation limit (QL) are the minimum detection and quantitation limits of the analyte in the sample, respectively, that can be reliably quantified. These values were measured using the standard deviation (SD) of the y-intercept (σ) and the slope of the calibration curve (S) derived based on the linearity evaluation and were calculated as follows: DL = 3.3 σ/S and QL = 10 σ/S. Precision is an analytical procedure used to evaluate repeatability and reproducibility. The repeatability of the experiment was assessed by measuring the intra- and inter-day precision. Three reference compounds were repeated five times (*n* = 5) at low, medium and high concentration levels (10.0, 100.0, and 300.0 µg/mL). Intra-day precision was injected on the same day, while inter-day precision was injected on days 1, 3, and 5. The results were determined using the relative standard deviation (RSD) of the peak area. The %RSD was calculated as follows: RSD (%) = (standard deviation/mean measured amount) × 100. %RSD value of <2% means that the developed HPLC-PDA method is precise and reproducible enough for simultaneous analysis and evaluation of the three compounds. A recovery test is conducted to verify the accuracy of the proposed method. %Recovery was determined by spiking each reference solution at three concentration levels (12.0, 60.0, and 120.0 µg/mL) into the WGM sample. The analysis was repeated three times (*n* = 3), and the measured values were calculated as follows: % recovery = (found concentration-original concentration)/spiked concentration × 100. Stability was used to evaluate the degree of change in the solution concentration with storage conditions and time. The stock solution of the WGM samples and the three reference compounds was maintained at room temperature (R.T., 22 ± 3 °C) and 4 °C for 72 h, and the measurements were taken five times (*n* = 5) at 6, 24, 48, and 72 h. The stability results were evaluated as the %peak area change and %RSD between the mean peak area of the control group (0 h) and that of the experimental group (6, 24, 48, and 72 h).

### 2.7. In Vitro Metabolic Inhibition Study in Rat Liver S9 Fraction

The composition of the reaction mixture used for in vitro CYP metabolic inhibition studies was as follows: 100 mM potassium phosphate buffer, 10 mM MgCl_2_, 1 mM NADPH, rat liver S9 fraction (1 mg/mL), CYP isoforms-specific probe substrate, and 50 μg/mL WGM. CYP isoforms-specific probe substrates were BUS for CYP3A, DIC for CYP2C, and DEX for CYP2D [34,35,37]. Dose–response curves assessed metabolic inhibition of BUS in the presence of 1 μM BUS and 10 different concentrations (0, 0.1, 0.5, 1, 2, 5, 10, 20, 50, and 100 μM) of 7-methoxyluteolin-5-*O*-glucoside, pilloin 5-*O*-β-d-glucopyranoside or rutarensin. The mixture without substrate and inhibitor was incubated for 5 min at 37 °C, after which the substrate and inhibitor were added to initiate the enzyme reaction [38,39]. After incubating BUS (reaction time: 30 min), DIC and DEX (reaction time: 60 min) at 37 °C at a speed of 500 oscillations/min, a sample was obtained from the reaction mixture and mixed with cold acetonitrile containing an internal standard (IS) to terminate the reaction. After sample preparation, the samples were analyzed using the ultra-performance liquid chromatography–tandem mass spectrometry (UPLC-MS/MS) method (see the sections entitled ‘Biological Sample Preparation’ and ‘UPLC-MS/MS Conditions’ in the Supplementary Materials).

### 2.8. In Vivo Pharmacokinetic Study in Rats

To evaluate the effects of WGM on the pharmacokinetics of BUS in vivo, 12 rats underwent surgical cannulation of the femoral artery after anesthetization via an intramuscular injection of 10 mg/kg zoletil [40,41]. After sufficient recovery from anesthesia, 6 rats were administered a single oral dose of BUS (30 mg/kg) and 6 rats were administered WGM (1 g/kg) together with BUS. Approximately 150 μL blood samples were collected from a cannula inserted into the femoral artery at 0, 2, 5, 10, 15, 30, 60, 120, 180, 240, 360, and 480 min post-administration. The blood was immediately centrifuged at 3000× *g* for 10 min at 4 °C to obtain the plasma [42], which was pretreated and analyzed using the UPLC-MS/MS method.

### 2.9. Pharmacokinetic Analysis and Statistic

The half-maximal drug inhibitory concentration (IC_50_) of the WGM marker compounds for the metabolism of BUS was determined using the four-parameter logistic equation indicated as follow (GraphPad Prism ver. 5.01; San Diego, CA, USA):

$$y=\mathrm{Min}+\frac{\mathrm{Max}-\mathrm{Min}}{1+({\frac{x}{{\mathrm{IC}}_{50}})}^{-P}}$$

where x is inhibitor concentration, y is response, Max is the initial y values, Min is the final y values, and P is the Hill coefficient. The average plasma pharmacokinetic parameters, including the area under the curve from time 0 extrapolated to infinity (AUC_inf_) and the terminal half-life (t_1/2_), were determined using a non-compartment model (WinNonlin Ver. 3.1; Certara, Inc., Princeton, NJ, USA) [43]. The peak time (T_max_) and maximal concentration (C_max_) were obtained directly from the observed plasma concentration-time profiles. A *t*-test was used to compare two unpaired means, and a *p*-value of below 0.05 was considered statistically significant.

## 3. Results and Discussion

### 3.1. Identification and Purification of Compounds

NMR spectroscopy was used to reveal the molecular structures of three compounds isolated from WGM, and their chemical shifts are as follows:

*7-Methoxyluteolin-5-O-glucoside* (**1**): C_22_H_22_O_11_; HR-ESI-MS [M − H]^−^ *m*/*z* 461; ^1^H NMR (DMSO-*d*_6_, 500 MHz): δ 7.42 (1H, d, *J* = 2.2 Hz, H-6′), 7.40 (1H, s, H-2′), 7.01 (1H, d, *J* = 2.4 Hz, H-8), 6.91 (1H, d, *J* = 2.4 Hz, H-6), 6.89 (1H, d, *J* = 8.1 Hz, H-5′), 6.60 (1H, s, H-3), 4.75 (1H, d, *J* = 7.5 Hz, Glc-1), 3.90 (3H, s, 7-OCH_3_), 3.80–3.10 (6H, m, Glc-2~6), ^13^C NMR (DMSO-*d*_6_, 125 MHz): δ 176.9 (C-4), 163.4 (C-7), 161.6 (C-2), 158.4 (C-9), 158.1 (C-5), 149.3 (C-4′), 145.7 (C-3′), 121.4 (C-1′), 118.6 (C-6′), 115.9 (C-5′), 113.0 (C-2′), 109.2 (C-10), 105.9 (C-3), 103.8 (Glc-1), 103.5 (C-6), 96.6 (C-8), 77.6 (Glc-5) 75.7 (Glc-3), 73.6 (Glc-2), 69.9 (Glc-4), 60.9 (Glc-6), 56.0 (7-OCH_3_).

*Pilloin 5-O-β-d-glucopyranoside* (**2**): C_23_H_24_O_11_; HR-ESI-MS [M − H]^−^ *m*/*z* 475; ^1^H NMR (DMSO-*d*_6_, 500 MHz): δ 7.52 (1H, dd, *J* = 8.5, 2.3 Hz, H-6′), 7.42 (1H, d, *J* = 2.3 Hz, H-2′), 7.07 (1H, d, *J* = 8.5 Hz, H-5′), 7.01 (1H, d, *J* = 2.4 Hz, H-8), 6.89 (1H, d, *J* = 2.5 Hz, H-6), 6.66 (1H, s, H-3), 4.74 (1H, d, *J* = 7.6 Hz, Glc-1), 3.88 (3H, s, 7-OCH_3_), 3.85 (3H, s, 4′-OCH_3_), 3.78-3.11 (6H, m, Glc-2~6), ^13^C NMR (DMSO-*d*_6_, 101 MHz): δ 176.9 (C-4), 163.6 (C-7), 161.2 (C-2), 158.5 (C-9), 158.2 (C-5), 150.9 (C-4′), 146.8 (C-3′), 123.0 (C-1′), 118.4 (C-6′), 112.9 (C-2′), 112.2 (C-5′), 109.3 (C-10), 106.5 (C-3), 104.1 (Glc-1), 103.5 (C-6), 96.5 (C-8), 77.6 (Glc-5), 75.8 (Glc-3), 73.6 (Glc-2), 70.0 (Glc-4), 61.0 (Glc-6), 56.1 (7-OCH_3_), 55.8 (4′-OCH_3_).

*Rutarensin* (**3**): ^1^H NMR (DMSO-*d*_6_, 600 MHz): C_31_H_30_O_16_; δ 8.04 (1H, d, *J* = 9.6 Hz, H-4′), 7.87 (1H, d, *J* = 6.4 Hz, H-4), 7.72 (1H, d, *J* = 8.5 Hz, H-5′), 7.28 (1H, s, H-5), 7.24 (1H, d, *J* = 2.5 Hz, H-8′), 7.23 (1H, s, H-8), 7.14 (1H, dd, *J* = 8.6, 2.5 Hz, H-6′), 6.39 (1H, d, *J* = 9.6 Hz, H-3′), 5.14 (1H, d, *J* = 6.8 Hz, Glc-1), 4.28 (1H, d, *J* = 11.7 Hz, Glc-6), 4.07 (1H, dd, *J* = 11.8, 6.8 Hz, Glc-6), 3.80 (3H, s, 6-OCH_3_), 2.60 (1H, d, *J* = 14.4 Hz, H-2‴), ^13^C NMR (DMSO-*d*_6_, 151 MHz) δ 172.61 (C-5‴), 170.52 (C-1‴), 160.04 (C-2′), 159.43 (C-7′), 156.86 (C-2), 155.03 (C-9′), 148.75 (C-9), 146.73 (C-6), 146.35 (C-4′), 144.12 (C-7), 137.17 (C-3), 129.99 (C-4), 129.86 (C-5′), 114.61 (C-10′), 114.06 (C-5), 113.71 (C-3′), 112.35 (C-6′), 109.60 (C-10), 104.43 (C-8′), 103.08 (C-8), 99.41 (Glc-1), 76.52 (Glc-2), 73.80 (Glc-5), 73.02 (Glc-3), 69.77 (Glc-4), 68.97 (C-3‴), 63.16 (Glc-6), 56.09 (6-OCH3), 45.26 (C-4‴), 45.13 (C-2‴), 27.54 (C-6‴).

**Figure 1:**
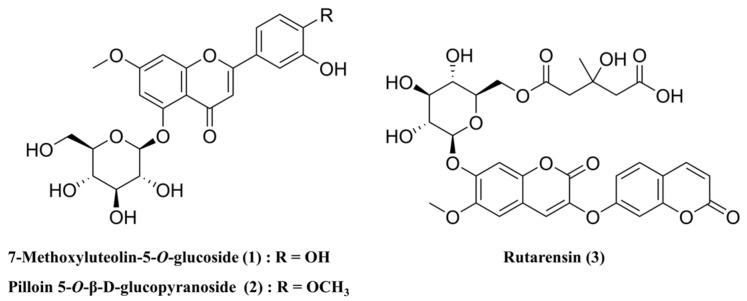
Chemical structures of the three compounds isolated from WGM.

Compared with the literature data, compound **1** was identified as 7-methoxyluteolin-5-*O*-glucoside (**1**) [44], compound **2** as pilloin 5-*O*-β-d-glucopyranoside (**2**) [1], and compound **3** as rutarensin (**3**) [45]. In Figure 1, the chemical structures of each component are shown. The isolated compounds were purified using semi-preparative HPLC. The purified compounds 7-methoxyluteolin-5-*O*-glucoside (**1**) (12.5 mg, purity ≥ 97%), pilloin 5-*O*-β-d-glucopyranoside (**2**) (16.2 mg purity ≥ 97%), and rutarensin (**3**) (56.2 mg, purity ≥ 98%) were used as reference samples.

### 3.2. Development of HPLC-PDA Conditions

**Figure 2:**
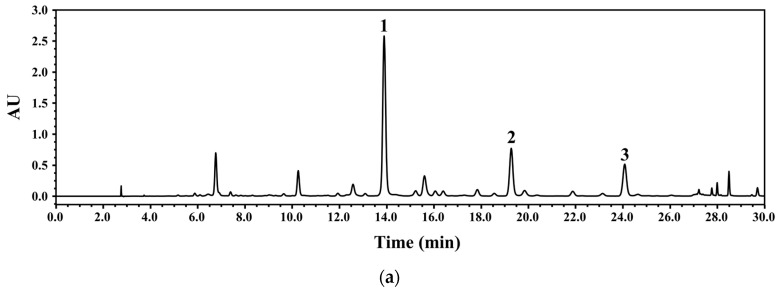

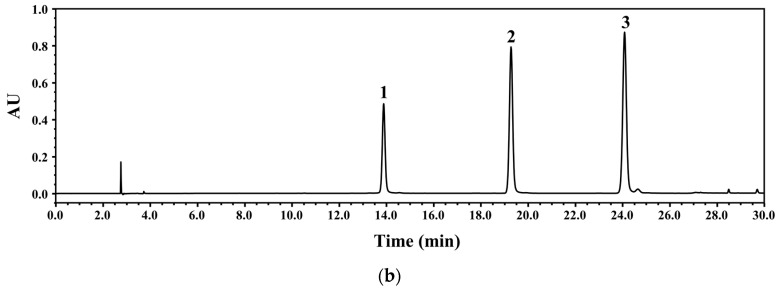
HPLC chromatogram (λ = 340 nm) of WGM (**a**) and reference mixture (**b**): 7-methoxyluteolin-5-*O*-glucoside (**1**), pilloin 5-*O*-β-d-glucopyranoside (**2**), and rutarensin (**3**).

HPLC-PDA was used to develop a method for simultaneous analysis of the three marker components isolated from WGM. The developed method should be able to rapidly analyze the target components with high resolution. Therefore, the analysis conditions were optimized by considering column temperature, mobile phase, gradient conditions, flow rate, and wavelength. For the mobile phase, 0.1% FA was added to acetonitrile (A) and water (B) to increase resolution. The gradients were first tested with the WGM sample solution under the conditions of 0–60 min and 5–95% A. When separation was performed under the first condition, the three marker components [7-methoxyluteolin-5-*O*-glucoside (**1**), pilloin 5-*O*-β-d-glucopyranoside (**2**), and rutarensin (**3**)] were detected. However, the retention time of 7-methoxyluteolin-5-*O*-glucoside (**1**) was relatively long (approximately 20 min), and pilloin 5-*O*-β-d-glucopyranoside (**2**) and rutarensin (**3**) could not be properly separated. To improve resolution and shorten analysis time, a multi-step gradient was applied, and the temperature (e.g., 30, 35, and 40 °C) and flow rate (e.g., 0.7 and 1.0 mL/min) were set. The analysis wavelength was selected by scanning the 210–400 nm range using PDA. Finally, a gradient elution system of 0.0–5.0 min, 13–20% A; 5–23 min, 20–30% A; 23–25 min, 30–70% A; 25–30 min, 70–70% A, was used. The optimized conditions successfully separated the three marker components of the WGM sample, which were detected within 30 min, as shown in Figure 2.

### 3.3. Validation of Methods

**Figure 3:**
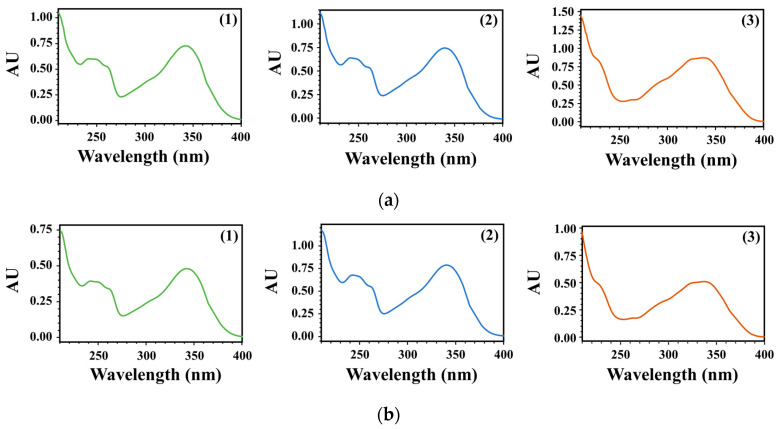
PDA spectra of WGM (**a**) and reference solutions (**b**): 7-Methoxylutolin-5-*O*-glucoside (**1**), pilloin 5-*O*-β-d-glucopyranoside (**2**), and rutarensin (**3**).

Specificity was evaluated by comparing the HPLC-PDA chromatograms obtained from the WGM samples and the reference mixture. In the WGM samples and reference mixture, the retention time (*t*_R_) and maximum absorption wavelength (λ_max_) of the three compounds were as follows: 7-Methoxyluteolin-5-*O*-glucoside (*t*_R_ of 13.886 min, and λ_max_ at 241.3 and 341.4 nm), pilloin 5-*O*-β-d-glucopyranoside (*t*_R_ of 19.280 min, and λ_max_ at 242.5 and 340.2 nm), and rutarensin (*t*_R_ of 24.088 min, and λ_max_ at 336.6 nm). In addition, as shown in Figure 3, the PDA spectra matched with each other. As a result, all parameters [retention time (*t*_R_), PDA spectra, and maximum absorption wavelength (λ_max_)] of the WGM samples and reference mixture were matched. Therefore, the specificity of the three compounds was validated, and the developed method allowed for the selective and accurate analysis of the analyte.

Linearity was verified by analyzing the three marker components in the concentration range of 5.00 to 600.00 μg/mL. Each reference compound was tested six times at each concentration to obtain calibration data at six concentrations. The regression equation and coefficient of determination (*r*^2^) for the calibration curve are listed in Table 1. The *r*^2^ values of all the curves showed good linearity, with *r*^2^ > 0.999 across the concentration range used.

The DL and QL were between 0.72–3.34 μg/mL and 2.19–10.12 μg/mL, respectively. The results for each component are shown in Table 1.

The intra- and inter-day %RSD for the three marker components at low, medium, and high concentrations ranged from 0.26–1.55% and 0.60–1.68%, respectively. Among the three marker components, the %RSD values for the intra- and inter-day of pilloin 5-*O*-β-d-glucopyranoside (**2**) were slightly higher. However, as shown in Table 2, the total repeatability of all compounds did not exceed 2%; thus, the precision was validated according to the ICH Q2(R1) guidelines.

To verify the accuracy of the method by evaluating the closeness of the measured values to the actual values, known concentrations of the three marker components were spiked into the WGM samples, and the %recovery was calculated using repeated (*n* = 3) measurements of the analyte. As shown in Table 3, the average %recovery for 12.0, 60.0, and 120.0 μg/mL of the three marker components was within 93.42–117.55%, and the %RSD values were below 2%. In other words, the %recovery was the lowest when the medium concentration of rutarensin (**3**) was spiked and the highest when the medium concentration of 7-methoxyluteolin-5-*O*-glucoside (**1**) was spiked.

The stability of the three marker components to storage conditions (R.T. and 4 °C) was evaluated by calculating the %difference and %RSD. Under all storage conditions, the three compound peak areas of the WGM sample solution increased after 6 h and decreased after 48 h. After 72 h, the peak areas of the three compounds increased by 0.99–3.56% from 0 h under all storage conditions. In addition, the %RSD was less than 2%, and stability was confirmed under all conditions. In the reference solution, the peak areas of the three compounds decreased after 6 h in 4 °C storage conditions. Under room temperature storage conditions, 7-methoxyluteolin-5-*O*-glucoside (**1**) and rutarensin (**3**) decreased by 0.54 and 3.23%, respectively, but pillion 5-*O*-β-d-glucopyranoside (**2**) increased by 1.71%. After 48 h, pillion 5-*O*-β-d-glucopyranoside (**2**) increased by 7.58 and 4.42% under conditions R.T. and 4 °C, respectively, showing an unstable state. Consequently, it is recommended to use the WGM sample and reference solution within 6 h after preparation and to store it at 4 °C. The stabilities of the three compounds in the WGM sample and reference solutions are detailed in Table 4.

### 3.4. Chemical Profiling of WGM

Quantitative analysis of the three marker components in the WGM samples was performed using the developed and validated HPLC-PDA assay. The assay was repeated five times (*n* = 5), and the content of each compound was calculated using a calibration curve. The three marker components in the WGM sample were measured to be within 17.10–51.81 mg/g. The most abundant marker compound in *W. ganpi* was 7-methoxyluteolin-5-*O*-glucoside (**1**) (Table 5).

### 3.5. Inhibitory Effects of WGM and Marker Compounds on Cytochrome P450 Activity in the Rat Liver S9 Fractions

**Figure 4:**
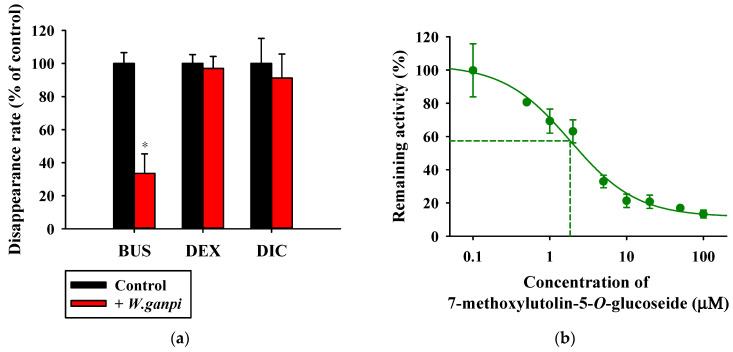

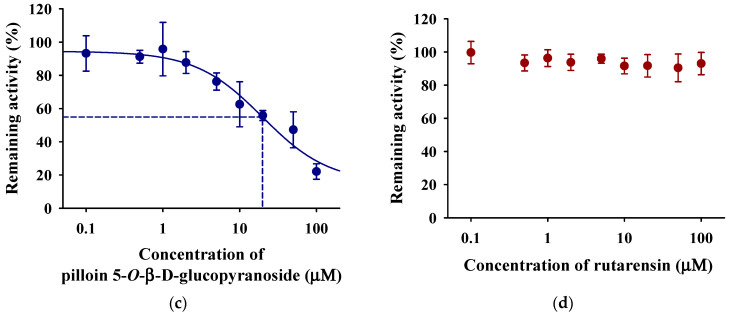
Effects of WGM on the disappearance rate of model cytochrome P450 substrate in rat liver S9 fraction (**a**). Dose–response curves for the inhibitory effect of 7-methoxylutolin-5-*O*-glucoside (**b**) pilloin 5-*O*-β-d-glucopyranoside (**c**), and rutarensin (**d**) on the disappearance of BUS in rat liver S9 fraction (*n* = 5). The asterisk represents a value significantly different from that of the other groups (*p* < 0.05).

The inhibitory effects of WGM on CYP activity were assessed by measuring the disappearance of various CYP substrates in the rat liver S9 fraction, as shown in Figure 4a. The metabolism of DEX and DIC did not change in the presence of 50 μg/mL WGM; however, it significantly inhibited the metabolism of BUS in the presence of 50 μg/mL WGM compared to the control group (*p* = 0.000169 for BUS). The inhibitory effect of CYP at various concentrations (0–100 μM) of WGM marker compound on metabolism of BUS in rat liver S9 fraction was evaluated through the construction of a dose–response curve (Figure 4b–d). 7-Methoxyluteolin-5-*O*-glucoside and pilloin 5-*O*-β-d-glucopyranoside, among the marker compounds of WGM, inhibited BUS metabolism in the rat liver S9 fraction with IC_50_ values of 2.73 and 18.7 μM, respectively. However, rutarensin did not inhibit BUS metabolism. These results indicate that WGM inhibits the metabolic activity of CYP3A but not CYP2C and CYP2D [37,46,47] and that the inhibition of CYP3A activity via WGM is attributed to 7-methoxyluteolin-5-*O*-glucoside and pilloin 5-*O*-β-d-glucopyranoside.

### 3.6. In Vivo Pharmacokinetic Studies in Rats

**Figure 5:**
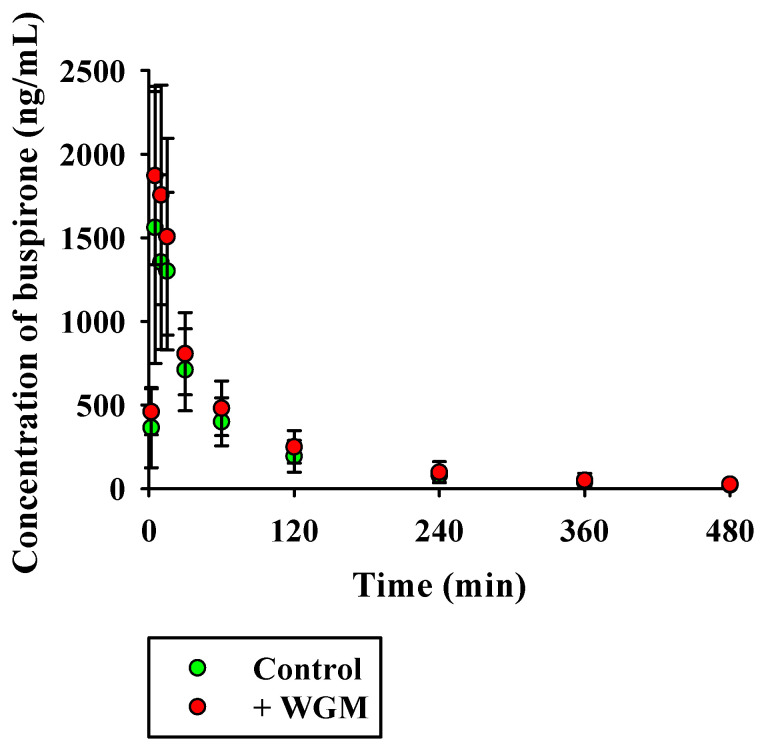
The mean plasma concentration-time curve of BUS after oral administration of 30 mg/kg in rats (green circles) or following co-administration with 1 g/kg WGM (red circles). Symbols show means and error bars represent SD (*n* = 6).

The mean plasma concentration versus time profiles following the oral administration (30 mg/kg) of BUS with or without concomitant oral administration of WGM (1 g/kg) in rats are shown in Figure 5. The corresponding pharmacokinetic parameters of BUS with or without WGM administration are presented in Table 6. The T_max_ of BUS was 5–15 min, showing rapid absorption into the systemic circulation. Compared to the control group, the WGM group did not show significant changes in the pharmacokinetic parameters of the administered BUS (*p* > 0.05). The inhibitory effects of WGM on various CYP metabolic activities were assessed by comparing the disappearance of the substrate (BUS, DEX, and DIC) between the absence and presence of 50 μg/mL WGM. Although the metabolic activity of BUS was significantly decreased in the rat liver S9 fraction, the activities of DEX and DIC did not significantly decrease in the presence of WGM. Among the marker components of WGM, 7-methoxyluteolin-5-*O*-glucoside and pilloin 5-*O*-β-d-glucopyranoside exhibited inhibitory effects on BUS metabolism in the rat liver S9 fraction with IC_50_ values of 2.73 and 18.7 μM, respectively. However, rutarensin did not inhibit BUS metabolic activity. This suggests that 7-methoxyluteolin-5-*O*-glucoside and pilloin 5-*O*-β-d-glucopyranoside regulate the metabolism of CYP3A [47,48]. Based on the results of this experiment, the pharmacokinetics were investigated after the oral administration of BUS with or without WGM in rats. The group administered simultaneously with WGM increased AUC_inf_ and C_max_ by 21.0% and 23.7% compared to the control group, but there was no significant difference between the two groups. BUS is primarily eliminated via hepatic metabolism in rats and humans [35]. In vitro CYP inhibition studies using rat liver S9 suggest that BUS, a substrate of CYP3A, reduced liver-specific clearance (CL_int,H_). However, there were no significant changes in the pharmacokinetic parameters of BUS in vivo with or without WGM, suggesting that there was no change in the liver-specific clearance of BUS. Several factors might have contributed to these findings. One of the causes may be low bioavailability due to chemical degradation of phytochemicals and the intestinal/hepatic first pass [38,41,49,50], and plasma and liver concentrations of inhibitors may be insufficient for the IC_50_ obtained in vitro CYP inhibition studies [51,52]. Consequently, a single oral dose of WGM did not have significant effects on the pharmacokinetics of buspirone in rats, suggesting that WGM cannot function as an inhibitor of CYP3A-mediated metabolism in vivo. This should consider the interspecies differences between rats and humans. Further studies are required to confirm whether WGM is a clinically relevant CYP3A4 inhibitor.

## 4. Conclusions

In this study, three marker components (7-methoxyluteolin-5-*O*-glucoside, pilloin 5-*O*-β-d-glucopyranoside, and rutarensin) were isolated from *W. ganpi* using the developed HPLC-PDA method. The developed analysis method was verified according to the analysis procedure outlined in the ICH Q2 (R1) guidelines, wherein 7-methoxyluteolin-5-*O*-glucoside, pilloin 5-*O*-β-d-glucopyranoside, and rutarensin can be used as marker components for the quality evaluation of *W. Ganpi*. In addition, we demonstrated that WGM inhibited the CYP3A-mediated metabolism of BUS in the rat liver S9 fraction. However, a single oral dose of WGM did not have significant effects on the pharmacokinetics of buspirone in rats, showing that WGM may not function as a CYP3A inhibitor in vivo. The present results are the first to report data regarding the in vitro and in vivo effects of WGM on CYP3A-mediated metabolism and pharmacokinetics of BUS in rats.

## Figures and Tables

**Table 1:** Linear range, regression equation, coefficient of determination, detection limit, and quantitation limit of three marker components (*n* = 6).

Compound ^a^	Linear Range (μg/mL)	Regression Equation ^b^	*r* ^2^	DL (μg/mL)	QL (μg/mL)
**1**	5.00–600.00	y = 20,102.01x + 91,314.67	0.9995	1.19	3.59
**2**	5.00–600.00	y = 18,769.28x + 9052.25	0.9995	0.72	2.19
**3**	5.00–600.00	y = 14,803.15x + 46,931.92	0.9995	3.34	10.12

^a^ 7-Methoxyluteolin-5-*O*-glucoside (**1**), pilloin 5-*O*-β-d-glucopyranoside (**2**), and rutarensin (**3**); ^b^ y = peak area (AU), x = sample concentration (μg/mL).

**Table 2:** Intra- and inter-day variabilities of three marker components.

Compound ^a^	Concentration (μg/mL)	Intra-Day (*n* = 5)		Inter-Day (*n* = 5)	
		Mean ± SD (μg/mL)	RSD (%)	Mean ± SD (μg/mL)	RSD (%)
**1**	9.70	10.6 ± 0.1	1.24	10.6 ± 0.1	0.94
	97.0	95.7 ± 0.5	0.47	96.2 ± 0.8	0.87
	291	291 ± 1	0.26	293± 4	1.43
**2**	9.70	9.40 ± 0.15	1.55	9.40 ± 0.10	1.11
	97.0	97.4 ± 0.4	0.45	97.5 ± 0.6	0.60
	291	291 ± 2	0.54	296 ± 5	1.68
**3**	9.80	11.2 ± 0.1	1.22	11.2 ± 0.1	0.78
	98.0	96.0 ± 0.5	0.51	97.1 ± 1.0	1.04
	294	295 ± 2	0.75	294 ± 2	0.80

^a^ 7-Methoxyluteolin-5-*O*-glucoside (**1**), pilloin 5-*O*-β-d-glucopyranoside (**2**), and rutarensin (**3**).

**Table 3:** Recoveries for three marker components of WGM (*n* = 3).

Compound ^a^	Original Conc. (μg/mL)	Spiked Conc. (μg/mL)	Found Conc. ± SD (μg/mL)	Recovery ± SD (%)	RSD (%)
**1**	804	11.6	817 ± 1	113 ± 2	1.86
		58.2	873 ± 1	118 ± 1	0.70
		116	915 ± 1	95.3 ± 1.3	1.39
**2**	196	11.6	208 ± 0	105 ± 1	0.98
		58.2	255 ± 0	100 ± 1	1.16
		116	311 ± 0	98.8 ± 0.5	0.48
**3**	173	11.8	183 ± 0	95.5 ± 1.6	1.68
		58.8	229 ± 0	93.4 ± 1.2	1.33
		118	285 ± 0	96.1 ± 0.3	0.36

^a^ 7-Methoxyluteolin-5-*O*-glucoside (**1**), pilloin 5-*O*-β-d-glucopyranoside (**2**), and rutarensin (**3**).

**Table 4:** Stabilities of three marker components in WGM and reference solutions (*n* = 5).

Solutions	Compound ^a^	Temp.	Peak Area (%)				RSD (%)
			6 h	24 h	48 h	72 h	
WGM	**1**	R.T.	100.80	100.10	97.43	100.99	1.33
		4 °C	100.10	98.87	98.63	101.60	1.28
	**2**	R.T.	101.41	101.08	98.97	103.35	1.52
		4 °C	100.32	99.18	98.86	102.02	1.34
	**3**	R.T.	101.30	101.14	98.98	103.56	1.58
		4 °C	100.19	99.30	98.91	102.15	1.38
Reference	**1**	R.T.	99.46	98.56	100.34	99.03	0.76
		4 °C	99.15	96.60	96.58	97.39	1.46
	**2**	R.T.	101.71	103.18	107.58	103.48	2.57
		4 °C	99.29	102.15	104.42	101.79	1.93
	**3**	R.T.	96.77	98.82	98.54	98.72	1.19
		4 °C	98.02	99.40	98.78	101.22	1.21

^a^ 7-Methoxyluteolin-5-*O*-glucoside (**1**), pilloin 5-*O*-β-d-glucopyranoside (**2**), and rutarensin (**3**).

**Table 5:** Amounts of three marker components in WGM (*n* = 5).

Compound ^a^	Amount		
	Mean (mg/g)	SD	%RSD
**1**	51.81	0.05	0.09
**2**	19.36	0.02	0.10
**3**	17.10	0.02	0.11

^a^ 7-Methoxyluteolin-5-*O*-glucoside (**1**), pilloin 5-*O*-β-d-glucopyranoside (**2**), and rutarensin (**3**).

**Table 6:** Pharmacokinetic parameters of BUS after oral administration of 30 mg/kg in rats or following co-administration with 1 g/kg WGM. Data are expressed as means ± SD (*n* = 6).

Parameter	BUS Alone	BUS with WGM
AUC_inf_ (μg·min/mL)	98.2 ± 39.7	119 ± 50
t_1/2_ (min)	111 ± 23	103 ± 27
C_max_ (ng/mL)	1629 ± 762	2016 ± 619
T_max_ (min)	10 (5–15)	5 (5–10)

## Data Availability

The data presented in this study are available upon request from the corresponding author.

## Supplementary Materials

The following supporting information can be downloaded at: https://www.mdpi.com/article/10.3390/nu15184061/s1. UPLC-MS/MS conditions; Biological Sample Preparation.
