# Supplementary material for: A High-Performance Liquid Chromatography with Photodiode Array Detection Method for Simultaneous Determination of Three Compounds Isolated from Wikstroemia ganpi: Assessment of the Effects on Cytochrome P450-Mediated Metabolism In Vitro and In Vivo
Source: Nutrients. 2023 Sep 19;15(18):4061. doi: 10.3390/nu15184061 (PMC10534693; doi:10.3390/nu15184061)
Supplement: Supplementary file 1 [file nutrients-15-04061-s001.zip › nutrients-2599123-supplementary.pdf]

Supporting Information

A High-Performance Liquid Chromatography with Photodiode Array Detection Method for Simultaneous Determination of Three Compounds Isolated from Wikstroemia ganpi: Assess-ment of the Effects on Cytochrome P450-Mediated Metabolism In Vitro and In Vivo

Min-Ji Keem [1,2,†], Seong-Wook Seo [2,3,†], Taeyoung Kim [2,3], Beom-Geun Jo [1,2], Su-Nam Kim [4], In-Soo Yoon [2,3,*] and Min Hye Yang [1,2,*]

[1] Department of Pharmacy, College of Pharmacy, Pusan National University, Busan 46241, Republic of Korea; mj_keem@pusan.ac.kr (M.-J.K.); bg_jo@pusan.ac.kr (B.-G.J.)

[2] Research Institute for Drug Development, Pusan National University, Busan 46241, Republic of Korea; mj_keem@pusan.ac.kr (M.-J.K.); sswook@pusan.ac.kr (S.-W.S.); taeyk@pusan.ac.kr (T.K.); bg_jo@pusan.ac.kr (B.-G.J.)

[3] Department of Manufacturing Pharmacy, College of Pharmacy, Pusan National University, Busan 46241, Republic of Korea; sswook@pusan.ac.kr (S.-W.S.); taeyk@pusan.ac.kr (T.K.)

[4] Natural Products Research Institute, Korea Institute of Science and Technology, Gangneung 25451, Republic of Korea; snkim@kist.re.kr (S.-N.K.)

[*] Correspondence: insoo.yoon@pusan.ac.kr (I.-S.Y.); mhyang@pusan.ac.kr (M.H.Y.)

[†] These authors contributed equally to this work.

Materials and Methods

UPLC-MS/MS Conditions

A Shimadzu UPLC-MS/MS system consisting of a pump (LC-30AD), an autosampler (SIL-30AC), a column oven (CTO-20AC), and a mass detector (LCMS-8050) was used (Shimadzu Co.). Liquid chromatographic separation of BUS, DEX, DIC and IS (internal standard; alpelisib 5 ng/mL) was conducted using a Poroshell 120 EC-C_{18} column (100 × 2.1 mm, 2.7 μm; Agilent) protected by a C_{18} guard column (SecurityGuard ULTRA; Phenomenex). Gradient elution of the mobile phase, consisting of 0.1% formic acid in deionized water (DIW; solvent A) and acetonitrile (solvent B), was performed at a flow rate of 0.3 mL/min at 40°C. The procedure was as follows (solvent A:solvent B, *v/v*): starting at 95:5 for 0 min, ramping from 95:5 to 15:85 for 3 min, ramping from 15:85 to 0:100 for 3 min, maintained at 0:100 for 1 min, returning to 95:5 for 0.01 min, and maintained at 95:5 min for 2.99 min (hence, the total run time was 10 min). The ion source parameters were set as follows: nebulizing gas flow, 3 L/min; drying gas flow, 10 L/min; heating gas flow, 10 L/min; interface temperature, 300°C; desolvation temperature, 250°C; and heating block temperature, 400°C. Signals of the precursor $[M+H]^+$ ion peaks were observed at *m/z* 386.20, 272.30, 297.65, and 442.10 for BUS, DEX, DIC, and IS, respectively. These precursor ions are further fragmented into product ions. The most abundant product ions were observed at *m/z* 122.15, 215.15, 214.15, and 328.00 for BUS, DEX, DIC, and IS. The optimized LC-MS/MS conditions resulted in retention times of 2.229, 2.321, 3.437, and 2.794 min for BUS, DEX, DIC, and IS.

Biological Sample Preparation

A measure of 50 μL of each biological sample, such as plasma, or S9 fraction, was

deproteinized with 200 μL of acetonitrile containing 5 ng/mL IS, vortex mixed for 5 min, and centrifuged at 16,000 × g for 10 min at 4°C. Then, 200 μL of the supernatant was collected, dried under vacuum using a SpeedVac (Eyela), reconstituted with 60 μL MeOH, and injected into the UPLC-MS/MS system.
